# Transition between Two Regimes Describing Internal Fluctuation of DNA in a Nanochannel

**DOI:** 10.1371/journal.pone.0016890

**Published:** 2011-03-15

**Authors:** Tianxiang Su, Somes K. Das, Ming Xiao, Prashant K. Purohit

**Affiliations:** 1 Department of Mechanical Engineering and Applied Mechanics, University of Pennsylvania, Philadelphia, Pennsylvania, United States of America; 2 BioNanomatrix Inc, Philadelphia, Pennsylvania, United States of America; Dalhousie University, Canada

## Abstract

We measure the thermal fluctuation of the internal segments of a piece of DNA confined in a nanochannel about 50
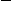
100 nm wide. This local thermodynamic property is key to accurate measurement of distances in genomic analysis. For DNA in 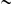
100 nm channels, we observe a critical length scale 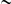
10 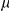
m for the mean extension of internal segments, below which the de Gennes' theory describes the fluctuations with no fitting parameters, and above which the fluctuation data falls into Odijk's deflection theory regime. By analyzing the probability distributions of the extensions of the internal segments, we infer that folded structures of length 150
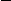
250 nm, separated by 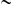
10 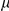
m exist in the confined DNA during the transition between the two regimes. For 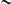
50 nm channels we find that the fluctuation is significantly reduced since the Odijk regime appears earlier. This is critical for genomic analysis. We further propose a more detailed theory based on small fluctuations and incorporating the effects of confinement to explicitly calculate the statistical properties of the internal fluctuations. Our theory is applicable to polymers with heterogeneous mechanical properties confined in non-uniform channels. We show that existing theories for the end-to-end extension/fluctuation of polymers can be used to study the internal fluctuations only when the contour length of the polymer is many times larger than its persistence length. Finally, our results suggest that introducing nicks in the DNA will not change its fluctuation behavior when the nick density is below 1 nick per kbp DNA.

## Introduction

Stretching DNA in nanochannels has emerged as an important technique for separating DNA, performing genome mapping, and also studying repressor-DNA interactions, *etc* [1]–[3]. On the other hand, DNA confined in nanochannels also serves as a simplified model for studying single polymer behavior in concentrated polymeric solutions and melts [4], [5]. For these reasons, mechanical behaviors of DNA inside nanochannels have attracted a long-standing interest. The two most well-known scaling theories in this field are those described by de Gennes [5] and by Odijk [6]. de Gennes' blob theory, which was later generalized by Schaefer and Pincus [7], assumes that the channel width 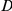 is much greater than the persistence length 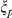 of the polymer. It models the moderately confined DNA as a chain of spherical blobs inside a cylindrical channel and gives the following expression for the end-to-end extension 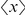 of the polymer [5], [7], [8]:
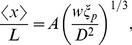
(1)where 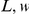 are the contour length and effective molecule width of the DNA respectively. The prefactor 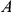 is found to be close to unity [9]. Odijk's theory, on the other hand, works for DNA under strong confinement in which 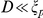. In this regime, the polymer is deflected back and forth by the channel walls and the end-to-end extension is predicted to be [6]:
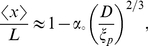
(2)where 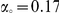 is a constant whose value was determined recently by simulations [10]. Aside from the scaling theories, Wang and Gao [11] showed that the end-to-end extension of a strongly confined polymer in the Odijk regime can be derived analytically by modeling the confinement effect as a quadratic potential 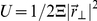. Here 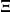 is the stiffness of the effective quadratic potential, which depends on the channel width 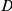, and 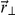 is the transverse displacement of the polymer from the axis of the nanochannel. Wang and Gao considered a confined chain under end-to-end applied force 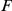 and obtained an expression for the total extension 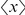 as a function of 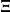 and 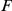. We set 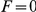 pN, substitute the relation between 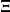 and 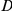 (see Supporting Information) into their expression, and find:
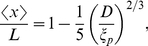
(3)which is the same as Eq.2, confirming the scaling theory of Odijk, and at the same time validating the use of quadratic confinement potentials in the strongly confined regime.

Both de Gennes' and Odijk's theories have been tested by experiments as well as simulations over the years [10], [12]–[16]. However, most of the studies so far have focused on the properties of the entire DNA, for example, the *end-to-end* extension 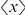, the corresponding *end-to-end* fluctuation 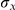, and also the relaxation time 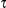 of the *entire* DNA *etc*. Local properties of a confined polymer, on the other hand, like the extension and fluctuation of its internal segments, are rarely investigated. In fact, local conformation and alignment of the confined DNA have been probed only recently [17], [18]. It is also not well understood whether the existing theories developed for an entire piece of DNA can be applied locally for its internal segments. These are important issues because, if one considers the case of genome mapping, it is the local fluctuation of the internal segments that determines the resolution of the mapping.

In this paper, we measure the longitudinal *internal* fluctuation of a piece of DNA confined in rectangular channels about 50
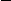
100 nm wide. We show that neither de Gennes' blob theory nor Odijk's deflection theory can completely describe the measured internal fluctuation versus mean extension profile. A critical length scale of 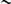
10 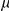
m for the mean extension is observed, below which the internal DNA segments are more ‘blob’-like, and above which Odijk's deflection theory works better. From the histograms of extension of the internal segments, we further infer that there exist folded structures of length 150
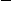
250 nm separated by 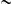
10 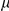
m along the backbone of the DNA during the transition between the two regimes. To justify the use of existing theories for studying the internal fluctuation, we focus on the Odijk regime and propose a method to explicitly calculate the internal fluctuation of a strongly confined DNA. We model the confinement effects by quadratic potentials and show that one can use the existing theories for end-to-end extension/fluctuation to describe the internal segments of the DNA when the contour length of the polymer is many times larger than its persistence length. Our model, which views the confined DNA as a discrete wormlike chain, can describe the fluctuations of heterogeneous polymers confined in non-uniform channels. It is also capable of capturing effects, like the influence of nicking sites on the DNA fluctuation profiles, which we will discuss at the end of the paper.

## Results and Discussion

**Figure 1:**
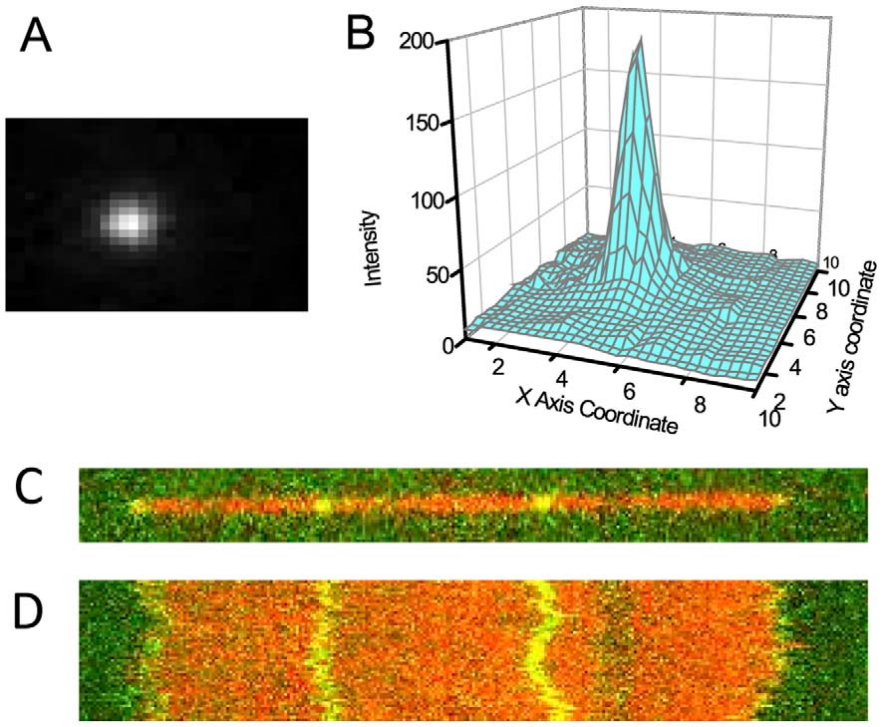
Measurement of the fluctuations of the internal segments of confined DNA. (A) Image of a dye label (Alexa-546) on a DNA backbone (backbone not shown) with 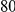 ms exposure time. (B) 2D surface plot of the raw image (intensity of the dye vs. the X Y coordinates). (C) Image of one T4 DNA fragment (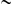
36 microns) with backbone (red) and internal labels (green). (D) Time series (8 seconds) of the DNA showing the fluctuations of backbone and internal labels. In (D), the red trace is the backbone and the green traces are the trajectories of internal dye labels.

To visualize the internal segments, dye-labeled (Alexa-546) nucleotides are introduced into the backbones of the nicked 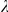 DNA (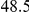 kbp, 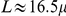
m), 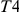 DNA (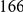 kbp, 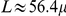
m) and bacterial artificial chromosome (BAC) human DNA clones (MCF7 BAC clone 9I10, fragmented, full length 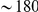
kbp, 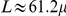
m) (Fig. 1) [19]. The DNA molecules are then driven by electric field into the nanochannels. With the Alexa-546 labels excited by light, extension of each internal segment is recorded frame-by-frame. Average extension 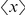 and the root mean square (rms) fluctuation 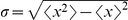 for each internal segmenet are calculated and plotted in the 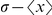 profile.

**Figure 2:**
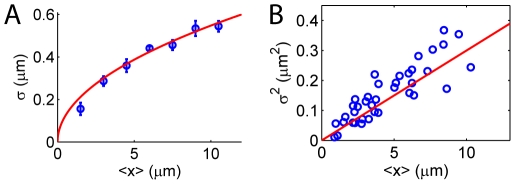
Internal fluctuation of 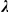 DNA confined in a 80 nm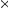 130 nm channel. (A) The measured rms fluctuation 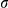 versus mean extension 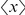 for the internal segments of the DNA agrees very well with de Genne's theory with no fitting parameters (red curve, Eq.4). (B) A linear 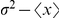 profile confirms the 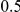 power law of 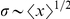 of the de Gennes' theory. Note, however, that here we have maximum 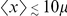
m. As shown in a subsequent figure (Fig. 4) and in the text, for longer polymer with a maximum 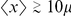
m, the data deviates significantly from de Gennes' theory and even the 0.5 power law is lost.

In Fig. 2, we first show the result for 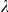 DNA confined in a 80 nm
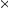
130 nm channel. The maximum 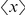, which is roughly the mean extension of the entire DNA, is about 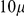
m, in agreement with the measurements of Tegenfeldt *et al* [12]. The internal fluctuation 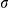 increases with 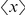 with a 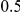 power law. This 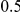 power law and even the magnitude of the fluctuation can be well captured by de Gennes' theory (discussed below) with no fitting parameters.

**Figure 3:**
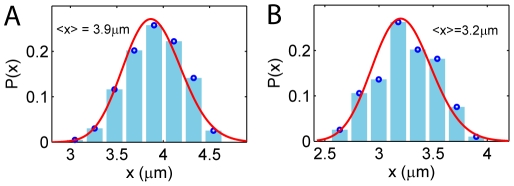
Probability distributions 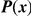 for 2 internal segments of 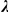 DNA inside a 80 nm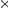 130 nm channel. The experimental data is fitted to Eq.7 (red). The fitting value 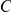 (Eq.7), when plugged back to Eq.6-2, recovers de Gennes's formula Eq.4.

The longitudinal fluctuation of the confined DNA in de Gennes' theory can be evaluated using the effective stiffness 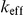 of the polymer: 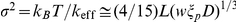 [12], [20]. Using this expression and Eq.1 to eliminate 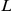, we get the relation between 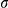 and 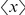:
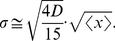
(4)Therefore, de Gennes' theory predicts a 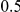 power law for the 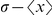 profile. It is interesting to note that the prefactor in Eq.4 depends only on the channel width 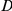, but not on the effective molecule width 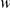, nor on the persistence length 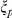. This implies that the 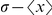 profile is independent of the ionic strength of the experimental buffer. To compare the theory with the measured internal fluctuation, we plot Eq.4 together with the experimental data in Fig. 2. Surprisingly, the data matches with the theory very well without any fitting parameters. Both the 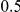 power law and the magnitude of the fluctuation are correctly predicted by Eq.4.

de Gennes' theory also gives the distribution of the extension 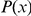, which we can compare to our measurement. We consider the recently proposed “renormalized” Flory-type free energy 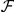 for a confined polymer [21] and its corresponding prediction of the longitudinal fluctuation:
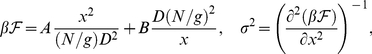
(5)where 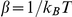, 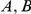 are two constants, 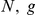 are the total number of monomers and the number of monomers inside a blob respectively [21]. Both of the relations can be rewritten in terms of 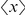 (which is the solution of 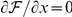) as:
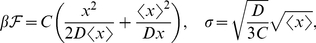
(6)with 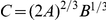 being a constant. The probability distribution 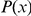 is therefore:
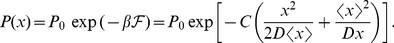
(7)Here 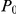 is a constant determined by the normalization condition. In our experiments, we record the extension 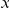 of each internal segment frame-by-frame and then calculate the distribution 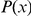 for *each* segment. Fig. 3 shows the measured 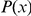 for two internal segments and their fitting results to Eq.7 (red). The result again implies that, for 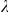 DNA confined in a 80 nm
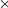
130 nm channel, the behavior of the internal segments can be well captured by de Gennes' theory. Moreover, by fitting the distribution 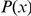 to Eq.7, we obtain the constant 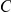, which, when plugged back into Eq.6-2, yields: 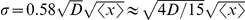 (here 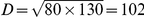 nm). Therefore, starting from the “renormalized” Flory-type free energy Eq.5, we recover Eq.4 with the same prefactor. This indicates that the prefactor in Eq.4 is quite accurate although it is derived from a scaling theory. It also explains why Eq.4 matches with the measured 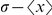 profile without any fitting parameters (Fig. 2). It is important to note that, for 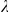 DNA confined in a 80 nm
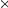
130 nm channel, the maximum 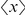 is less than 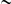
10 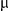
m (Fig. 2). We shall show next that for longer DNA whose maximum 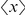 is greater than 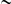
10 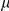
m, the measurement no longer agrees with de Gennes' theory. In particular, the 0.5 power law in the 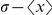 profile is lost.

**Figure 4:**
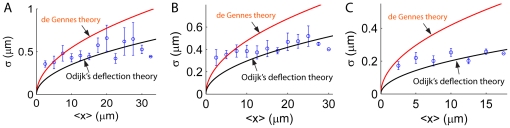
Fluctuation of the internal segments of (A) T4 DNA in 80 nm 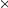 130 nm, (B) T4 DNA in 60 nm 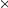 100 nm and (C) 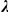 DNA in 50 nm 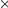 70 nm channels. For all cases, the maximum mean extension 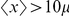
m. For (A) and (B), the data 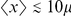
m agrees with de Gennes's theory (red, no fitting parameters). Deviation from de Gennes' theory begins at a critical 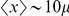
m, above which the data falls into the black curve predicted by the deflection theories of Odijk [6], Wang and Gao [11]. For tighter channels (C), the transition occurs earlier with most data falling in the deflection regime.

Fig. 4A shows the 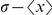 profile for the internal segments of T4 DNA in a 80 nm
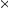
130 nm channel. The maximum 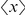, which is roughly the mean extension of the entire DNA, is about 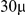
m, in agreement with the simulation result of Jung *et al* [14]. Fitting of 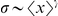 to the experimental data yields 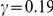, which is very different from the prediction of de Gennes' theory (Eq.4). Similar results are found for DNA in channels of different sizes: 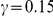 for T4 DNA confined in 60 nm
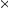
100 nm channels (Fig. 4B) and 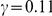 for 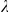 DNA in 50 nm
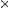
70 nm channels (Fig. 4C). In all these cases the maximum 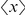 is greater than 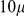
m. We note, however, that in Fig. 4, the experimental data for segments with 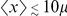
m still matches with de Gennes' theory (except for the 50
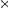
70 nm channel case, which we will explain later). It is the data with 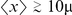
m that deviates significantly from de Gennes' prediction. In fact, if we plot the fluctuation results for short segments with 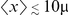
m for 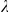 and T4 DNA together, the two profiles are almost identical, satisfying de Gennes' theory (see Supporting Information Fig. S1).

**Figure 5:**
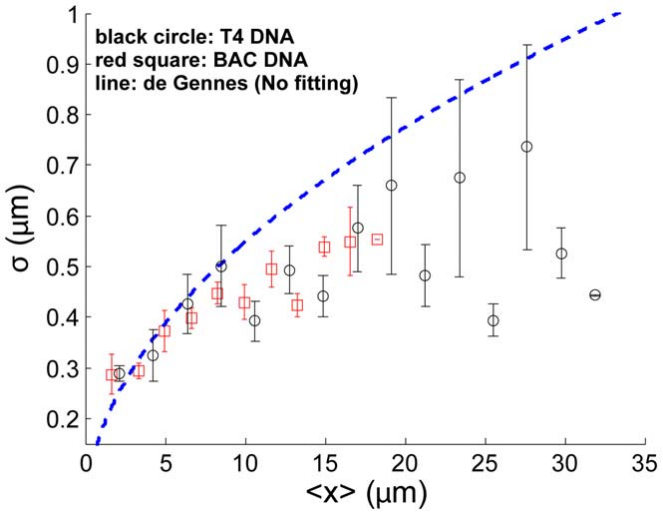
Internal fluctuation 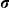 versus mean extension 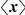 for BAC (red squares) and T4 DNA (black circles) in a 80 nm 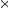 130 nm channel. This figure shows that DNAs from two different sources give almost identical results, which suggests that agreement with de Gennes theory for short internal segments, and deviation from de Gennes' theory for long internal segments, are both sequence independent.

To rule out the possibility that the observed difference between 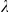 DNA and T4 DNA stems from sequence variations, we perform the same experiments on the bacterial artificial chromosome (BAC) human DNA clones (MCF7 BAC clone 9I10), which also has maximum 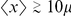
m. As shown in Fig. 5, the results for the BAC DNA are almost identical to those for the T4 DNA. In particular, for small 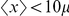
m, both match with de Gennes' prediction without any fitting parameters, while for 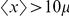
m, both identically deviate from de Gennes' prediction. This suggests that the deviation from de Gennes' theory for long internal segments truly stems from segment size, not from sequence variations.

**Figure 6:**
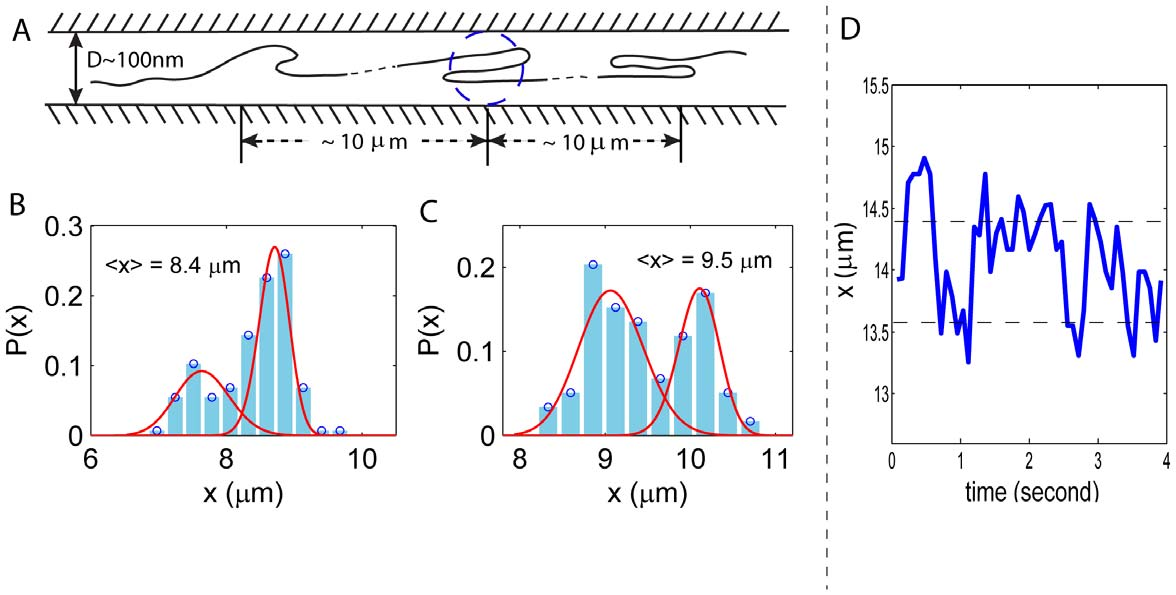
(A) Folded structures in the backbone of confined DNA. Each branch of the structure is about  nm, about the width of the channel size. The structures are separated by a distance 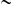
10 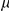
m. (B, C) Distribution of extension 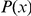 for 2 internal segments that contain the folded structures. In disagreement with de Gennes' prediction, the distributions show 2 peaks, from which we infer the existence of the folded structures. However, the structures are not stable as the two peaks in the distributions are comparable in height. The red curves fitted to the left peaks on the histogram are from de Gennes' theory (Eq.7) and the ones superimposed on the right peaks are from the deflection theory (Eq.10). (D) Extension 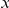 versus time for a single internal segment that shows two peaks in the distribution 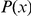. The extension of this particular internal segment seems to fluctuate around two values shown by the dashed lines. This gives rise to the two peaks seen in the probability distribution.

To better understand the deviation from de Gennes' prediction, we further look i nto the local structures of the confined DNA. Odijk showed recently that even in a 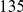 nm channel, DNA can fold back on itself, giving rise to a global persist ence length much larger than 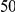 nm, the intrinsic persistence length of the DNA [18], [22]. Because of this, Odjik argued that the transition from Odijk's regime to de Gennes' regime could be delayed with the increase of the channel size [18]. To check whether such local folded structures exist in the DNA in our experiments, we measure the extension distribution 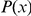 for each single internal segment (see “Materials and Methods” for details). We find that for most internal segments whose mean extension is longer than 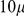
m, the distribution 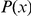 shows two or more peaks (Fig. 6B–C). From this observation, we infer that there indeed exist some folded structures in those internal segments – one peak in the distribution corresponds to the folded configuration, and the second peak corresponds to the extended configuration (Fig. 6). The existence of folded structures can be also inferred from the typical extension 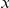 versus time plot as shown in Fig. 6D, where the steps in 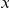 correspond to different states of the internal segments. Furthermore, we find that in the distribution 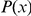, the measured distances between any two peaks are always integral multiples of 400
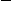
500 nm, indicating that the difference in extension of a single folded structure and its extended form is about 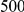 nm, ten times the persistence length of the DNA. This further implies that each branch of the folded structure is about 150
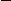
250 nm, if we assume each folded structure has two (loop) or three (hairpin) branches (Fig. 6). Also, by checking the location of the internal segments that show multiple-peak distributions, we find that the folded structures are separated by 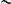
10 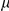
m, which roughly agrees with the value of 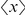 above which de Gennes' theory fails to match with the experimental data (Fig. 4). In the following we show that for 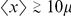
m the fluctuation data is better described by Odijk's deflection theory.

To exactly (rather than in a scaling sense) evaluate the fluctuation of DNA in the Odijk deflection regime, we extend the theory recently developed by Wang and Gao [11]. This theory represents the DNA as a strongly confined wormlike chain (fluctuating elastic rod) subjected to an additional end-to-end force 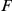 and produces the relation between the mean extension 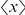 and 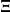, the stiffness of the effective confinement potential (which is a function of the channel width 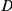):
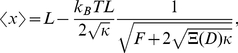
(8)where again, 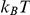 is the thermal energy, 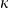 is the bending modulus of the polymer, and in a rectangular channel the stiffness of the confinement potential can be expressed as 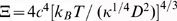, with 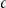 being a constant. Using Eq.8, we calculate the effective stiffness of the DNA as 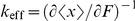, and then evaluate the fluctuation as 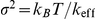:
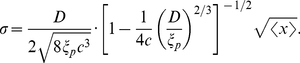
(9)Leaving 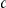 as a free parameter, we fit Eq.9 to the experimental data with 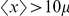
m in Fig. 4A–C (black curves) and obtain 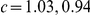 and 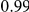 respectively. For the BAC DNA confined in 80 nm
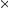
130 nm channels shown in Fig. 5, we obtain 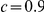 from a similar fit. The fact that all the four sets of experimental data for different channel widths yield the same 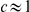 makes sense because 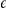 is expected to be a universal constant independent of 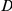. Moreover, the constant 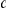 comes from the expression for the free energy of confined chains in the Odijk regime and it has been estimated by Burkhardt to be 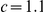 [23], which is very close to our fitting results. This strongly suggests that in the large mean extension regime 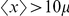
m, the DNA segments are better described by the deflection theory.

Furthermore, from Fig. 4A to C, we observe that the length of the error bars decreases with the decrease of the channel size. The reason for this may be that for moderately confined DNA, the local folded structures can form and unravel with comparable rates, as indicated by the similar height of the two peaks in the distribution in Fig. 6B–C. Therefore, the behaviors of the confined polymer is a competition between de Gennes' type and Odijk type regimes and the error bar is large. As the channel size becomes smaller, Odijk's theory begins to dominate, resulting in smaller error bars.

**Figure 7:**
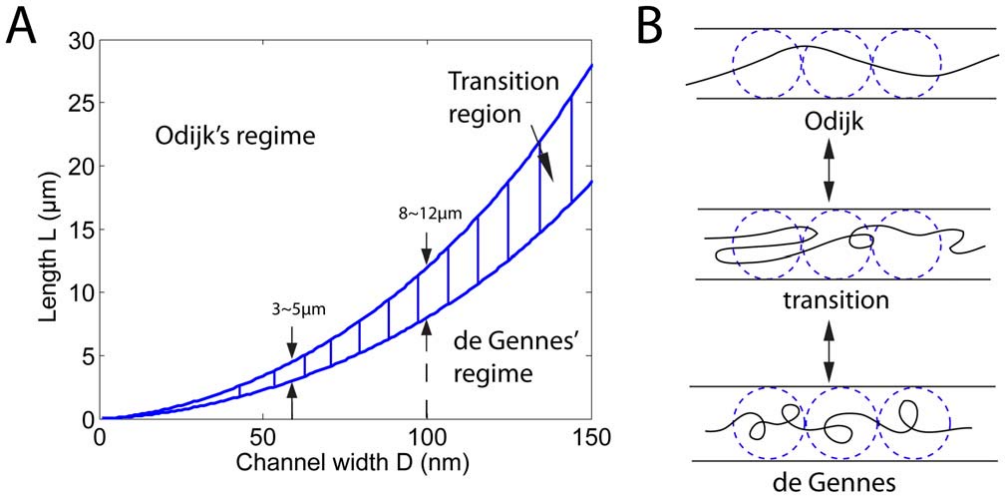
(A) Phase diagram showing two regimes on the 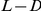 plane, assuming 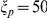 nm for DNA. Transition from de Gennes' to Odijk's regime can occur when 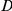 decreases with 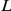 fixed, or when 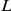 increases with 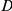 fixed. (B) DNA with local folded structures as an intermediate state between de Gennes's and Odijk's regimes. In experiments, we observe heterogeneity in the intensity profile of YOYO-1 dye along the backbone of a confined DNA, which suggests the existence of the local folded structures (see Supporting Information Fig. S2).

By integrating the force-extension relation Eq.8, we obtain the free energy expression 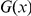 in the Odijk (or Wang and Gao) deflection regime (see Supporting Information), which further leads to the distribution for the extension 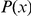:
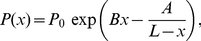
(10)where 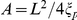, 
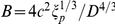
 and 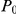 is the normalization factor. We fit this expression to the right peaks in Fig. 6B–C and find that reasonable parameters (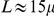
m, 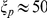 nm) give excellent matches with the measured probability distributions in experiments. In fact, we can use this free energy expression to understand the transition from a different point of view. We note that the internal segments are expected to stay in the regime with lower free energy, and that regime transition occurs when the free energies in the two regimes are equal. By comparing the free energies in the two regimes, we draw a phase diagram on the 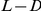 plane in Fig. 7. The result shows that as 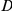 decreases, the transition length 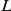 decreases. Theoretically, the phase diagram involves an undetermined constant, which we fit such that transition occurs in the range 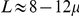
m when 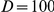 nm. Then the result shows that at 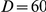 nm, the transition length is 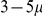
m, which roughly agrees with our experimental result for 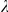 DNA in a 50 nm
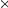
70 nm channel (Fig. 4C). The phase diagram shows that transition from de Gennes' to Odijk's regime can occur when 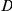 decreases with 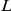 fixed, or when 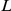 increases with 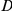 fixed.

We also measure the end-to-end extension for DNA with different lengths (longer than 10 microns) in a 60 nm
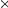
100 nm channel and the result agrees with Odijk's theory (Fig. S3).

In the above analysis, we have applied the theories (de Gennes, Odijk, Wang and Gao) for the *end-to-end* extension/fluctuation to evaluate the *internal, or local* extension/fluctuation of a confined DNA. The assumption behind this is that when the internal segments are much longer than the persistence length of the DNA, the behavior of the segments is not very different from that of the entire DNA (with the same length) because the boundary conditions do not play a significant role [24]–[26]. To verify such an assumption, we explicitly calculate the internal fluctuation in Odijk's regime by extending a theory we developed earlier [26], and then compare our results to the theories developed for an entire piece of DNA.

**Figure 8:**
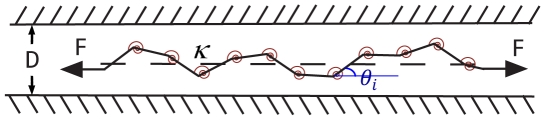
Discrete wormlike chain model for confined DNA in a nanochannel. The confined wormlike chain, subjected to and end-to-end applied force in general, has bending energy represented by a spring of stiffness 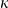 at each node.

Following the procedure in ref.[26], we model the polymer as a confined discrete 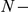
segment wormlike chain, or fluctuating elastic rod (Fig. 8). The Hamiltonian consists of 3 terms (Eq.11): (1) bending energy, (2) confinement energy, and (3) potential energy of an end-to-end applied force as shown in Fig. 8.
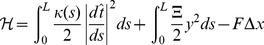
(11)

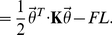
(12)In the bending energy term, 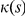 is the bending modulus of the DNA and it can vary along the arc length 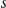 so that the polymer is not necessarily homogeneous in mechanical properties. 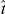 is the tangent vector along the polymer. For the confinement potential term, we follow Wang and Gao's approach [11] and use an effective quadratic energy characterized by the coefficient 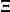, with 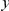 being the transverse displacement. In general, 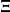 can be a function of the arc length 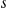 in case the confinement is not uniform. Also, for 3D chains in rectangular channels, 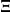 can be different in the two transverse directions. For the potential energy term, we consider the chain subjected to an end-to-end force 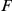, which can be set to zero if no force is applied. 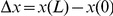 is the end-to-end extension of the chain. Up to a second order approximation, the Hamiltonian can be written in matrix form as shown in Eq.12, with 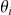 being the discretized tangent angles and 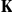 being the 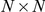 stiffness matrix of the chain [26].

It has been shown that when there are no constraints on twist (as is the case here), thermodynamic properties of a 3D chain can be easily generated from those of two 2D chains [26]. Therefore, for simplicity, here we describe the theory for 2D chains and plot the results for the corresponding 3D chains.

**Figure 9:**
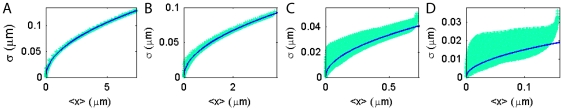
Fluctuation versus mean extension of internal segments of the strongly confined DNA in 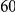 nm channels (Eq.13 and Eq.14). The contour lengths of the DNA are (A) 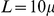
m, (B) 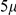
m, (C) 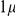
m and (D) 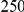 nm. For a long DNA (A and B), data from internal segments of various locations of the chain collapse on the a curve with 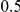 power law (light green). The result agrees with Eq.9 (blue), which is derived for the end-to-end fluctuation of a confined DNA. For short DNA however (C and D), no power law is found as data from various locations of the chain do not collapse onto a single curve (light green). Therefore, formulae derived for the end-to-end fluctuation of the confined DNA, such as Eq.9 (blue), cannot be used for internal fluctuation. The boundary effect is so significant that the rms fluctuation 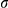 not only depends on 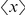, but also on the location of the internal segments.

**Figure 10:**
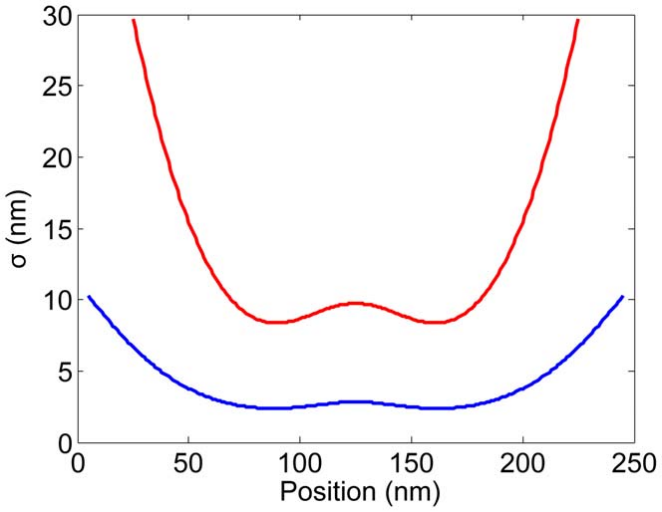
Fluctuation as a function as the position of an internal segment for a short chain. The contour length of the entire chain is short (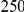 nm), so that the fluctuation not only depends on the length of the internal segment, but also on its position. Here we plot the fluctuation versus position for internal segments with the same size: 50 nm (red) and 10 nm (blue). For the internal segments close to the boundaries, the fluctuation is larger because they have more freedom compared to the segments inside the chain.

To get the internal fluctuation, we first need to calculate (1) the partition function, and (2) the angle fluctuation 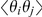. These are evaluated in the “Materials and Methods” section. Finally, for any internal segment between node 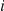 and node 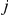 of the discrete chain, the mean extension 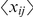 and the corresponding rms fluctuation can be explicitly calculated as:
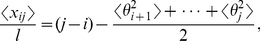
(13)

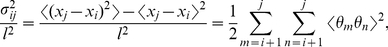
(14)where 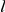 is the segment length of the discrete chain. In Fig. 9, we consider DNA in 60 nm
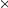
60 nm channels and plot 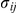 versus 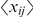 for all the pairs of internal nodes 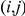 and see if the profiles match with the theories developed for the entire piece of DNA. Fig. 9(A) shows the result for a chain with contour length 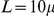
m, which is much larger than its persistence length 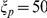 nm. The internal fluctuation profile agrees exactly with Eq.9, which is derived for the end-to-end fluctuations. In particular, all the data collapses into a single curve with 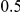 power law. As the contour length of the polymer decreases, however, (Fig. 9B–D), the internal fluctuation profile begins to scatter around the curve for the end-to-end fluctuation. This implies that, for short chains, the magnitude of internal fluctuation can be different, even if two internal segments have the same mean extension. The magnitude of the fluctuation depends strongly on where the internal segment is located. In fact, we show in Fig. 10 that the internal segments located at the two boundaries have larger fluctuation because they have more freedom to fluctuate compared to the segments inside the chain. The strong boundary effects on short chains (such as, DNA with contour length 0.6–7 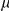
m) have been discussed by several groups recently [24]–[26]. Our results suggest that the accuracy of DNA sizing depends on the DNA contour length. For a short DNA with contour length 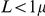
m confined in a 60 nm
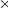
60 nm channel, the uncertainty of the measurement will be high. For the experimental results we discussed earlier, the 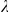 DNA, T4 DNA and BAC DNA all have contour lengths of tens of microns, for which boundary effects can be neglected. Therefore, it is safe to use the formulae for end-to-end extension/fluctuation to estimate the internal properties of the confined DNA in our experiments.

**Figure 11:**
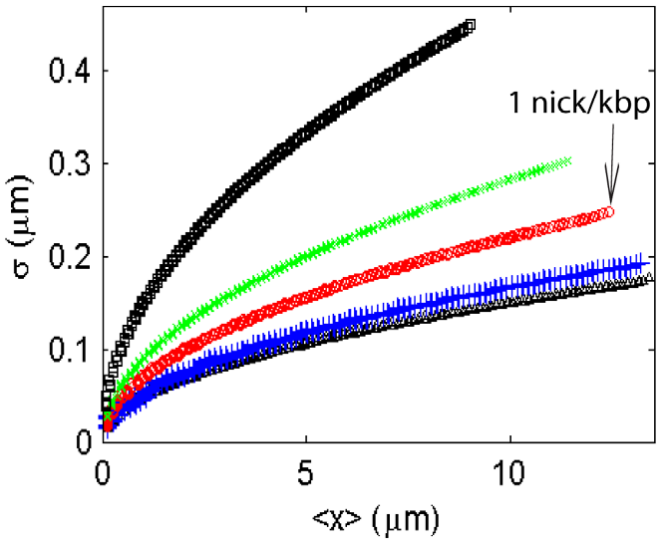
Fluctuation of a 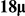 m long chain with persistence length 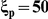 nm confined in a 60 nm 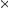 60 nm channel. From bottom to top: (1) 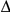: no nicks; (2) 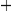: 10 nick in 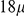
m; (3) 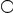: 50 nicks in 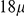
m; (4) 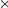: 100 nicks in 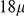
m; (5)□ : 200 nicks in 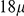
m. This figure shows that when the density of nicks is lower than 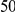 nicks per 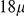
m, or 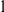 nick per kbp of DNA, the fluctuation profile is almost the same as that for a chain without nicks.

To measure the internal fluctuation, we have introduced nicks into the DNA so that internal sites along the DNA can be labeled. Since the theory discussed above allows for arbitrary bending modulus 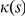 as a function of the arc length 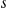, we can model the effect of nicking by setting 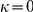 on some nodes of the discrete chain and see whether the nicks have significant effects on the behavior of the DNA. For simplicity, we assume here that the nicks are equally spaced along the chain. Fig. 11 shows that the fluctuation profile does not significantly deviate from the homogeneous chain with uniform 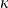 when there are less than 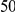 nicks along a 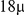
m chain (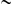
50 kbp DNA in a 60 nm
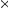
60 nm channel). In our experiments, the fluorescent tagging is introduced at the nicking endonuclease recognition sequence sites, which have much lower density than 1 nick/kbp in 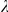, T4 and BAC DNA. Therefore, the nicks will not significantly affect the DNA internal fluctuation.

To summarize, in this paper, we have investigated the thermal fluctuations of the internal segments of a piece of confined DNA in a nanochannel. The channel size is on the order of the persistence length of the DNA and we have compared the fluctuation data to several theories in literature. We have found that for channel widths on the order of 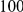
nm there exists a critical length scale 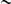
10 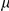
m for the mean extension of an internal segment below which the de Gennes' theory describes the internal fluctuations and above which the data agree better with Odijk's deflection theory. For long DNAs confined in nanochannels we have inferred that there are folded structures whose branches are about 3 times the persistence length of DNA which are separated by segments with mean extension 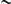
10
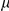
m. We surmise that these folded structures are indicative of a transition from the Odijk regime, in which the DNA is relatively straight, to the deGennes regime, in which the DNA is more blob-like. We have also presented a more detailed theory based on small fluctuations and incorporating the effects of confinement. We have shown that one can use the existing theories for end-to-end extension/fluctuations to study the statistical properties of internal segments only when the contour length of the chain is much larger than the persistence length of the molecule so that boundary effects play no role. Our calculations suggest that introducing nicks into the DNA can change its fluctuation behavior if the density of nicks is greater than about 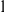 nick per kbp DNA.

## Materials and Methods

### Sequence specific labeling and DNA staining

In a 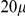
l reaction native, duplex DNA samples 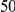 ng/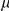
l (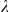, T4 DNA and also MCF7 BAC clone 9I10) are incubated with 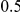 U of Nb.BbvCI (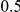 U/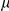
l) (NEB, Ipswich, MA) in 1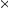 NEB buffer 2 (NEB) for 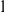 hr at 37

C and 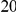 min at 65

C. The nicked DNA samples (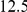 ng/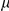
l) are then incubated for 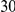 min at 50

C in 1
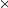
NEB thermopol buffer with DNA polymerase Vent (exo-) (NEB) at 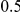 U/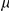
l in presence of a mixture of 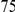 nM dAGC and 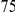 nM Alexa-546 labeled dUTP. Then, the DNA (4 ng/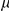
l) samples are stained with intercalating dye YOYO-1 iodide at 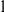 dye molecule per 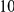 base pairs of DNA (Invitrogen Inc, Carlsbad, CA) in presence of 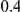 M DTT (Promega Inc, Madison, WI).

### Loading DNA into nanochannels

Fabrication of silicon based nanochannel chips has been described elsewhere [27], [28]. The DNA sample is diluted by 2 times using the flow buffer consisting of 1
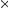
TBE, 3.6% Tween, and 10% Polyvinylpyrrolidone (PVP). Ultrapure distilled water is used for making solutions (Invitrogen Corp., Ultrapure water). The DNA molecules are driven by electric field (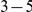 V) at the port of entrance of the chip and allowed to populate there for 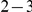 minutes [29]. Under higher voltage (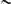
10 V), the populated molecules are moved to the locos and then through the micro pillar structure of the chip to convert from a compact globular conformation to an open relaxed one. At the 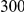
nm channel area the molecules adopt a more relaxed linear form with some heterogeneity on the backbone. With one end entering the nanochannel under the electric field, the DNA molecules elongate to a linear conformation with almost homogeneous backbone. Most of the structural heterogeneity progressively disappears as it interacted with the nanochannels, adopting fully confined equilibrium conformation after the field is off (relaxation time 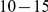 s). A buffer consisting 0.5
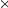
TBE, 1.8% Tween 20, 5% PVP has been used to flow the DNA molecules resulting in a stretch of 65%.

### Microscopy and image processing

The epi-fluorescence imaging is done in Olympus microscope (Model IX-71, Olympus America Inc, Melville, NY) using a 100
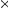
SAPO objective (Olympus SApo 100X/1.4 oil). YOYO-1, the DNA backbone staining dye (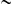
491 nm absorption, 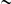
509 nm emission) is excited using 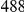 nm laser (BCD1, Blue DDD Laser Systems, CVI Melles Griot, Rochester, NY) whereas Alexa-546 (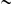
550 nm absorption, 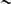
570 nm emission) is excited using 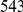
nm green laser (Voltex Inc, Colorado Springs, CO). The same filter cube consisting triple band dichroic and dual band pass emission filters (Z488/532/633rpc, z488/543 m respectively) (Custom made, Chroma Technology Corp. Rockingham, VT) is used for detection of YOYO-1 and Alexa-546 emission by alternative laser excitation (using external laser shutters, Thorlabs, Newton, NJ). The emission signal is magnified 1.6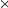 and detected by a back-illuminated, thermoelectric cooled charge coupled device (EMCCD) detector (iXon) (Andor, Ireland). About 200 sequential images of the labeled DNAs confined in nanochannels are recorded at 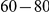 ms exposure time in blue-green alternative laser excitation.

### Recording and calculations

The intensity profile 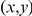 of each Alexa-546 label is fitted by a 2D Gaussian function to determine the position of the label 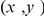 in the channel (Fig. 1B). The position of each internal label is followed frame-by-frame at a time interval of about 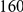 ms. The probability distribution, the mean value and the corresponding standard deviation of the distance between each pair of internal labels are calculated.

### Partition function and angle fluctuation

The partition function for a confined DNA, whose Hamiltonian is expressed in Eq.12, is: 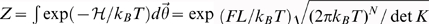, where 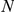 is the number of segments in the discrete chain. The angle fluctuation or correlation is the Boltzmann weighted average of 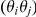 over all the configurations [26], [30]:
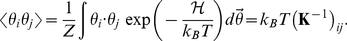
(15)Using Eq.15, we can explicitly calculate the mean extension and fluctuation of the internal segments (Eq.13–14).

## Supporting Information

Figure S1

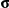
 **versus** 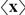 **profile for the** 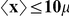 **m region.** Fluctuation of short internal DNA segments from different sources matches with de Gennes' theory with NO fitting parameters.(TIF)

Figure S2(A) The backbone intensity images of a confined DNA fragment (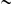
34 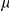
m) stained with YOYO-1 iodide in a 80 nm
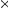
130 nm channel. The images are recorded at time interval of 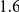 s. From the heterogeneity of the intensity profile, we infer that there exist some local structures on the backbone. (B) Images of the time series (8 seconds) of a T4 DNA fragment (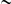
32 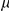
m). The backbone of the DNA is shown in red and the internal dyes are shown in green. The region with high fluorescence density is the area with local folded structures. The green traces are the trajectories of internal dye labels in the time series. This image shows two internal dyes coming together, which is evidence of formation of local folded structures.(TIF)

Figure S3
**Mean end-to-end extension** 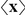 **versus contour length** 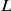 **of confined DNA in a 60 nm** 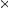 **100 nm channel.** The fitting result is 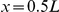, which is consistent with the prediction of the Odijk deflection theory: 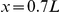.(TIF)

Text S1 (PDF)
