# Supplementary figures and images for: Transition between Two Regimes Describing Internal Fluctuation of DNA in a Nanochannel
Source: PLoS One. 2011 Mar 15;6(3):e16890. doi: 10.1371/journal.pone.0016890 (PMC3057976; doi:10.1371/journal.pone.0016890)

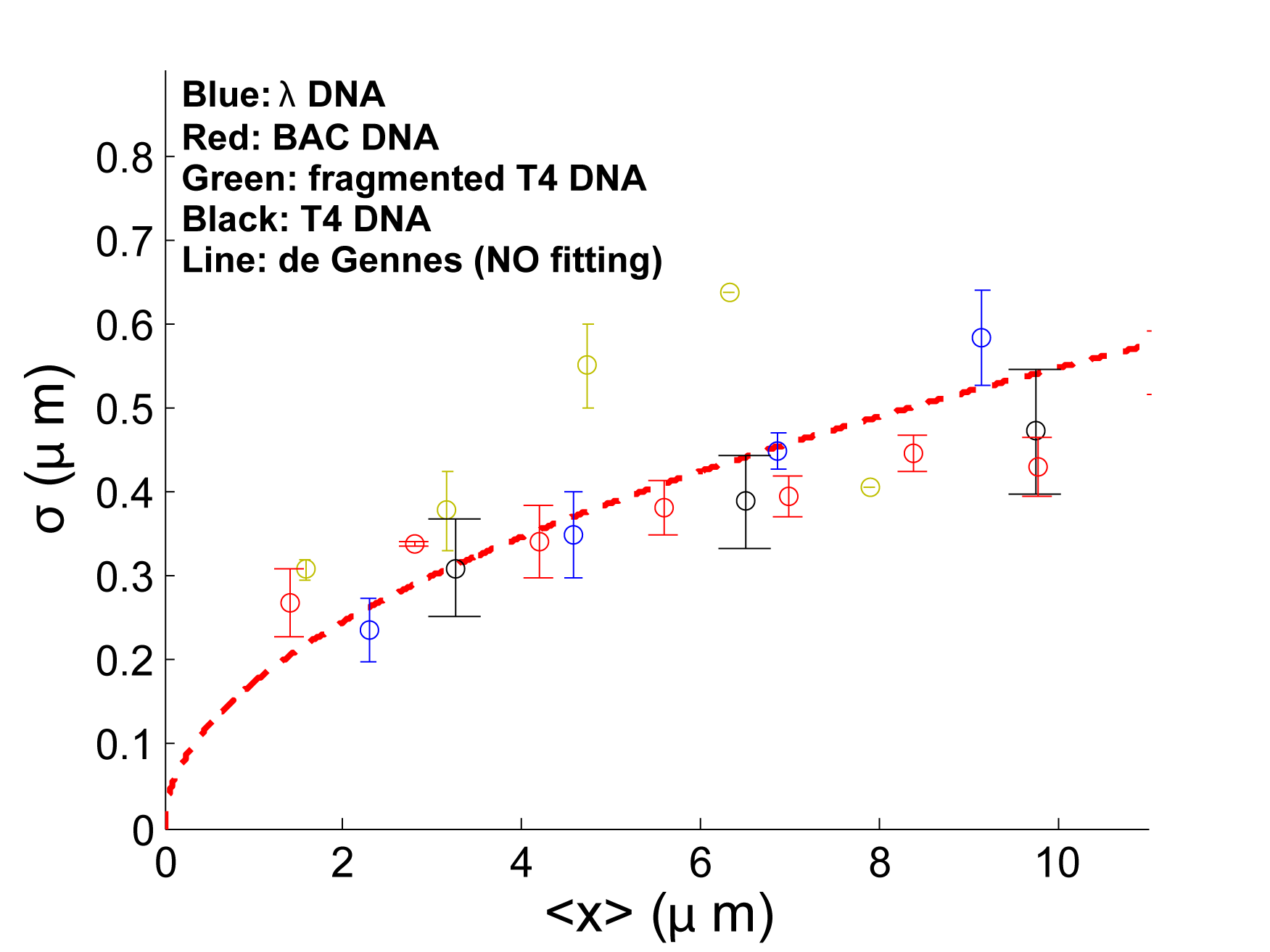

Supplement: Figure S1 — versus profile for the m region. Fluctuation of short internal DNA segments from different sources matches with de Gennes' theory with NO fitting parameters. (TIF) [file pone.0016890.s001.tif]

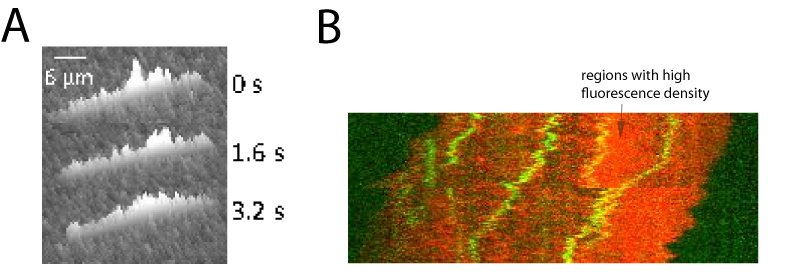

Supplement: Figure S2 — (A) The backbone intensity images of a confined DNA fragment (34 m) stained with YOYO-1 iodide in a 80 nm130 nm channel. The images are recorded at time interval of s. From the heterogeneity of the intensity profile, we infer that there exist some local structures on the backbone. (B) Images of the time series (8 seconds) of a T4 DNA fragment (32 m). The backbone of the DNA is shown in red and the internal dyes are shown in green. The region with high fluorescence density is the area with local folded structures. The green traces are the trajectories of internal dye labels in the time series. This image shows two internal dyes coming together, which is evidence of formation of local folded structures. (TIF) [file pone.0016890.s002.tif]

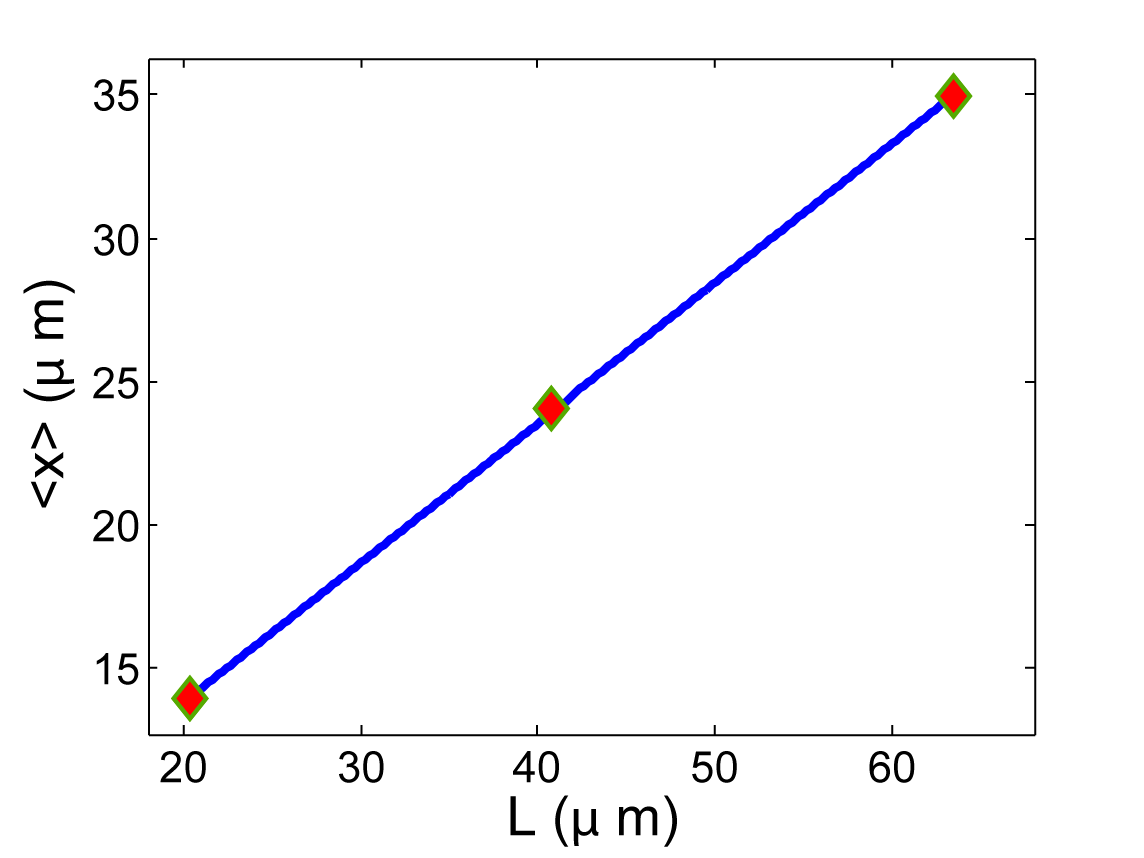

Supplement: Figure S3 — Mean end-to-end extension versus contour length of confined DNA in a 60 nm 100 nm channel. The fitting result is , which is consistent with the prediction of the Odijk deflection theory: . (TIF) [file pone.0016890.s003.tif]
