# Supplementary material for: Transition between Two Regimes Describing Internal Fluctuation of DNA in a Nanochannel
Source: PLoS One. 2011 Mar 15;6(3):e16890. doi: 10.1371/journal.pone.0016890 (PMC3057976; doi:10.1371/journal.pone.0016890)
Supplement: Text S1 — (PDF) [file pone.0016890.s004.pdf]

Supporting Information: Transition between two regimes describing internal fluctuation of DNA in a nanochannel

Tianxiang Su*, Somes K. Das†, Ming Xiao†, Prashant K. Purohit*

* Department of Mechanical Engineering and Applied Mechanics, University of Pennsylvania, Philadelphia, PA 19104, USA.

† Bionanomatrix Inc, 3701 Market Street, 4th floor, Philadelphia, PA, 19104, USA.

1 End-to-end extension $\langle x \rangle$ versus channel width D relation for Wang and Gao's theory

For a strongly confined DNA under force F, the total extension $\langle x \rangle$ as a function as F and Ξ (stiffness of the effective confinement potential) is found to be [1]:

$$\langle x \rangle = L - \frac{k_B T L}{2\sqrt{\kappa}} \frac{1}{\sqrt{F + 2\sqrt{\Xi(D)\kappa}}}, \tag{1}$$

where κ is the bending modulus of the polymer, Ξ relates to the channel width D in the following way:

$$\Xi = \frac{c^4}{4} \left(\frac{k_B T}{\kappa^{1/4} D^2} \right)^{4/3}, \tag{2}$$

and $c = 2.5$ is a constant for a cylindrical channel [1]. Setting $F = 0$ and plugging Eq.2 into Eq.1, we obtain:

$$\langle x \rangle = L \left[1 - \frac{1}{5} \left(\frac{D}{\xi_p} \right)^{2/3} \right]. \tag{3}$$

2 Fluctuation for short internal segments

The fluctuation for short internal segments is expected to be in the de Gennes' moderately confined regime. In Fig. S1, we plot the internal fluctuation profiles for short segments with $\langle x \rangle < 10\mu$m for 4 different sets of DNA: (1) λ DNA, (2) T4 DNA, (3) fragmented T4 DNA and (4) BAC DNA. Note that here we not only have short DNA, like λ DNA, but also long DNA like T4 and BAC DNA, but we discuss only the short internal segments on them in this section. The results for all the 4 sets of DNA are almost identical, and they all match with de Gennes' theory with NO fitting parameters. This result suggests that for all internal segments with $\langle x \rangle \leq 10\mu$m, irrespective of the sequence and length, de Gennes' theory works.

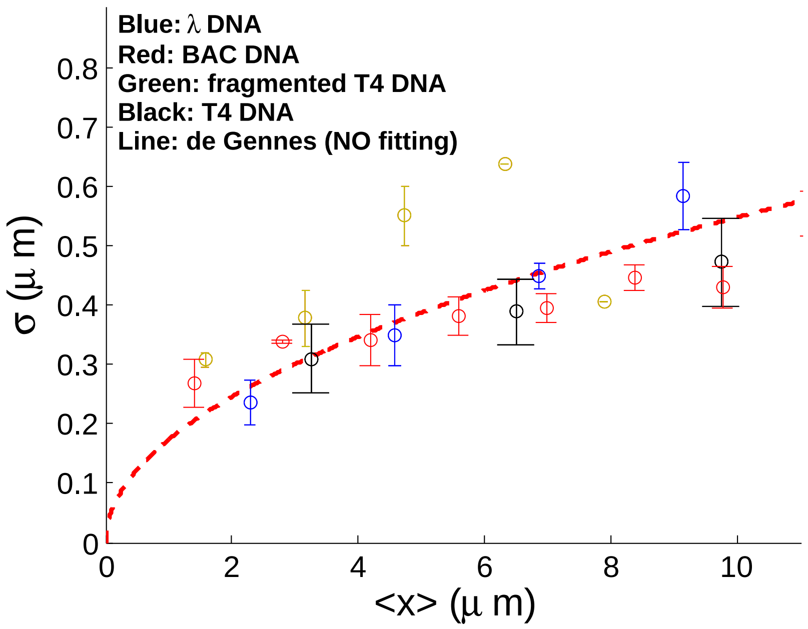

Figure S1:

3 Heterogeneity on the backbone of DNA

In experiments, we observe heterogeneity in the intensity profile of the YOYO-1 dye along the backbone of the DNA (Fig.S2), which can be evidence for the formation of local folded structures. For the left figure of Fig.S2, in the intensity profiles corresponding to 0s and 1.6s, localized peaks are clearly apparent (Fig.S2). These localized peaks could represent the deGennes' blobs or local folded structures. On the other hand, regions of uniform intensity could correspond to DNA in the Odijk regime. Heterogeneity in the DNA backbone fluorescence intensity is also shown in the right figure of Fig.S2. This figure shows two internal labels coming together, which is evidence for formation of local folded structure. Although these images gives us a visual picture of DNA confined to a nanochannel, we believe that the two-peak probability distributions shown in the main text provide much stronger evidence of the transition between the deGennes' and Odijk regimes than the fluorescence intensity profiles.

4 Total extension versus L relation

As another evidence that the deflection theory works for segments with $\langle x \rangle \gtrsim 10\mu$m, we measure the end-to-end extension for DNA with different lengths (but with mean end-to-end extension greater than 10 microns) in a 60nm×100nm channel and plot the result against the contour length (Fig.S3). A linear relation is found with a fitting result of $\langle x \rangle = 0.5L$. This is consistent with the deflection theories (formulae shown below), which, with numerical

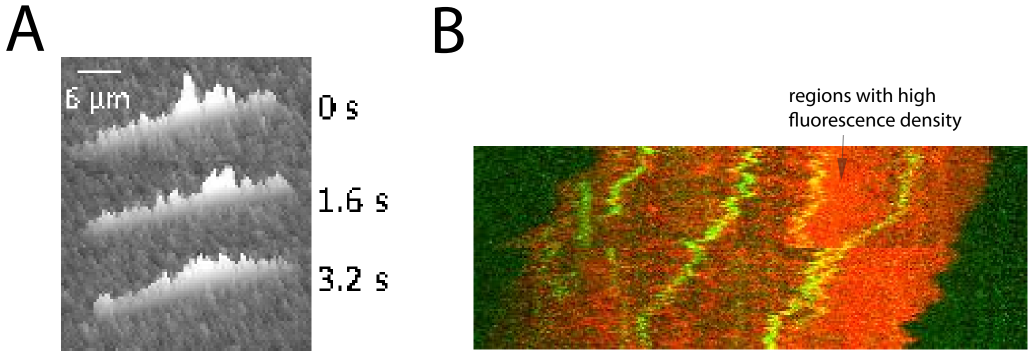

Figure S2:

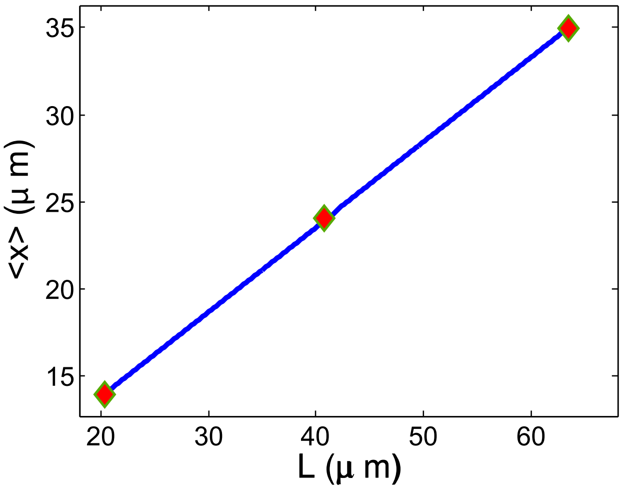

Figure S3:

values plugged in, gives $\langle x \rangle \approx 0.7L$.

$$\frac{\langle x \rangle}{L} \approx 1 - \alpha_\circ \left(\frac{D}{\xi_p}\right)^{2/3}, \quad \alpha_\circ = 0.17, \qquad \text{(Odijk, [2])} \tag{4}$$

$$\frac{\langle x \rangle}{L} = 1 - \frac{1}{5}\left(\frac{D}{\xi_p}\right)^{2/3}, \qquad \text{(Wang and Gao, [1])} \tag{5}$$

5 Distribution of extension in the deflection regime

The force-extension relation for a 3D confined chain in Odijk's regime is given in Eq.1 [1].

Since $dG = -xdF$, we can integrate Eq.1 to obtain the free energy $G = G(F,T)$:

$$\begin{aligned} G &= -\int xdF & (6) \\ &= -\int \left[L - \frac{k_B T L}{2\sqrt{\kappa}} \frac{1}{\sqrt{F + 2\sqrt{\Xi\kappa}}} \right] dF & (7) \\ &= -\frac{(k_B T L)^2}{4\kappa} \frac{2x - L}{(L-x)^2} + \text{const} & (8) \end{aligned}$$

This is the free energy in a fixed force ensemble, i.e $G(F,T) = E - TS - Fx$. We need the free energy in a fixed extension ensemble. Therefore:

$$\begin{aligned} G(x,T) &= G(F,T) + Fx & (9) \\ &= \frac{(k_B T L)^2}{4\kappa} \frac{1}{L-x} - 2\sqrt{\Xi K} x + \text{const} & (10) \end{aligned}$$

Denote $\rho = x/L$, then the free energy is:

$$\frac{G(\rho,T)}{k_B T} = \frac{A}{1-\rho} - B\rho + \text{const}, \quad (11)$$

where

$$A = \frac{L}{4\xi_p}, \quad B = \frac{4c^2 \xi_p^{1/3} L}{D^{4/3}}, \quad c = 1.1 \quad (12)$$

Therefore, the probability distribution is:

$$P(\rho) = P_0 \exp\left[B\rho - \frac{A}{1-\rho} \right] \quad (13)$$

with A, B given in Eq. 12 and P_0 being the normalization constant.

References

[1] Wang J, Gao H (2007) Stretching a stiff polymer in a tube. *J Mater Sci* 42:8838-8843.

[2] Odijk T (1983) On the statistics and dynamics of confined or entangled stiff polymers. *Macromolecules* 16:1340-1344.
